# Essential Oils for the Conservation of Paper Items

**DOI:** 10.3390/molecules28135003

**Published:** 2023-06-26

**Authors:** Felicia Menicucci, Eleonora Palagano, Marco Michelozzi, Andrea Ienco

**Affiliations:** 1 Institute for the Chemistry of Organo Metallic Compounds, National Research Council of Italy (CNR), Via Madonna del Piano 10, Sesto Fiorentino, 50019 Florence, Italy; andrea.ienco@iccom.cnr.it; 2 Institute of Biosciences and Bioresources, National Research Council of Italy (CNR), Via Madonna del Piano 10, Sesto Fiorentino, 50019 Florence, Italy; eleonora.palagano@ibbr.cnr.it (E.P.); marco.michelozzi@cnr.it (M.M.)

**Keywords:** essential oils, volatile organic compounds, paper-based objects, paper conservation, plant compounds, biodeteriogens, fungi

## Abstract

Archival documents and artworks stored in libraries frequently undergo degradative processes promoted by the so-called “biodeteriogens” that inhabit these places. A renewed interest in plant-derived products has arisen in those research groups focusing on cultural heritage preservation and looking for new and safe disinfection techniques. In this view, essential oils (EOs) and their volatile organic constituents are very appealing thanks to their versatility of action. A literature survey of the scientific publications involving EOs and/or their major constituents related to the conservation of paper items of cultural heritage interest is presented here, aiming to reveal benefits and limitations of such peculiar plant-derived compounds.

## 1. Plant Scented Arsenal Transferred into Archives

Also known as “essences”, essential oils (EOs) are complex aromatic mixtures composed of terpenes, terpenoids, phenols and other organic compounds of plant’s secondary metabolites. EOs are produced by several plants, with the aromatic and officinal EOs playing a major role. Secretory cells, canals and cavities are storage sites for the accumulation of these secondary metabolites, which are particularly concentrated in young tissues, that protect those parts typically exposed to predators [1,2,3]. Their synthesis is indeed strictly related to defense mechanisms of which a plant disposes, as these substances display well-known bioactivities of relevance from an ecological point of view, e.g., antibacterial, antifungal, antiviral and insect repellent activity [1,2,3,4,5].

The role of these compounds is, nevertheless, not only restricted to defense: for instance, flower and fruit scents are volatile attractive signals for many pollinators and seed dispersers, thus favoring plants’ reproductive success [2,6]. Characterized by intense smell and high volatility, EOs unsurprisingly find their primary traditional application in perfumery, though recently their use has been expanded to many other sectors, such as food packaging, cosmetics and agricultural technology, to name but a few [6,7,8,9]. Such an arsenal of versatile substances represents a treasure trove for those research fields and industries geared towards the design of non-synthetic innovative products. In this context, EO experimentation also finds a place in the preservation of cultural heritage, with the majority of the investigations focusing on paper, stone and wood [10,11,12,13,14,15,16,17].

Regarding this paper, it represents the main support that humankind has been exploiting to store and pass down knowledge; therefore, its preservation is extremely relevant [18,19]. Likewise, for other organic materials, the deterioration of documents and paper artworks of historical interest is unavoidable, though it can be controlled and strongly limited via careful conservation and restoration work. Cleaning and disinfection methods are also essential to prevent takeover by microbial communities, insects and other detrimental biodeteriogens, and new, safe and non-invasive techniques are urgently needed in a sector where toxic substances have been largely used for decades [20].

The first paper attesting the use of thymol for paper conservation dates back to 1986 [21], though the majority of the papers addressing the use of EOs in the field of archival material conservation have been published from 2012 until today, inspiring the present survey (Table 1) [10,11,12,13,22,23,24,25,26,27,28,29]. An overview of these works is presented in the following paragraphs, resuming the current state of-the art and highlighting benefits and limitations of plant EOs and their main constituents in the preservation of paper-based items. 

Briefly, in the second section of this review, we explain the importance of providing detailed composition information about EO mixtures, showing how GC-MS analysis may represent the tool of choice to reach this purpose. The third section focuses, firstly, on fungi, which are the leading agents promoting the degradation processes of paper items, and, secondly, on the main *in vitro* tests used to assess EOs’ bioactivity. In addition, this section reports the most frequently experimented EOs and/or single terpene constituents for the treatment of cellulose-based materials. Section 4 shows some case studies of paper items disinfected with EO constituents, whereas section number five deals with the structural analysis and the most useful parameters that are considered for assessing any possible alteration in a paper-based material subjected to an EO-based treatment. Finally, the last section presents the EO-derived products tested in the treatment of paper items to date, showing how resorting to technology may represent a worthy pathway to follow to overcome certain inherent boundaries of EOs and VOCs, such as their poor water solubility and high volatility.

## 2. EO Mixtures and Their Different Degrees of Bioactivities

The scientific literature contains several studies reporting no or only generic information about the chemical composition of the EOs used for experimentations. Each EO is a complex mixture of constituents characterized by a specific chemical composition, which directly influences its degree of effectiveness [3]. This observation means that changing the relative ratios of these constituents may create mixtures displaying completely different levels of efficacy; therefore, providing complete information about the composition of an EO is of extreme importance.

In a mixture, there are two or three prevailing components—known as volatile organic compounds (VOCs)—which, generally, define the set of bioactivities of an EO, and gas chromatography-mass spectrometry (GC-MS) analysis is the tool of choice to finely characterize the chemical composition of its volatile fraction [1,30]. This technique enables the determination of well-defined chemical profiles (chemotypes) that can be used as a fingerprint to identify and distinguish a specific mixture from another one. For instance, Borrego et al. [22] and Tomić et al. [29] tested the antimicrobial activity of different Eos, and both studies used oregano EO from *Origanum vulgare*. It is worth noting that the main EO constituents detected by Tomić [29] were γ-terpinene (19.6%), carvacrol (15.6%), p-cymene (11.0%) and sabinene (8.8%), whereas the terpene composition determined by Borrego [29] was completely different, containing thymol (38.0%), cis-β-terpineol (16.5%), terpinen-4-ol (10.2%), γ-terpinene (7.3%), α-terpinene (4.3%), p-cymene (3.7%), sabinene (3.7%) and carvacrol (3.4%).

Few of the works reviewed in this paper reported detailed EO composition [22,26,29], whereas partial or no information was provided by the majority of these studies.

Karbowska-Berent et al. [12] compare the effect of three different substances—tea tree oil, ethanol and hydrogen peroxide— that were tested in the disinfection of a series of paper-based historical items, and ascribes the damages caused by the treatment with tea tree oil to a variety of non-identified organic substances that comprise this EO, thus recommending not using it on paper items.

Another paper examined the degradative activities of two filamentous fungi, i.e., *Scopulariopsis* sp. and *Fusarium* sp., which are able to adhere to paper and were hazardous agents of mycosis [31]. Their response to the treatment with thyme and oregano EOs was investigated *in vitro*. Interesting results were obtained from these tests in terms of antifungal activity, even though only one very high concentration was tested, which completely prevented the growth of both fungi. Therefore, no comparison between the inhibitory effect exerted by the two EOs was performed, and neither of their chemical compositions were reported [23].

Providing detailed information about the EOs used in experiments would be of great relevance to understanding the complex mechanisms of action regulating their degree of effectiveness, as well as to comparing the efficacy of different mixtures, in order to select the most suitable option for the intended purpose. Complete composition information would also help us to cross-compare results obtained from similar investigations, increasing the scientific knowledge of these substances.

## 3. Paper Biodeteriogens, *In Vitro* Tests and Most Recurrent Substances

**Figure 1:**
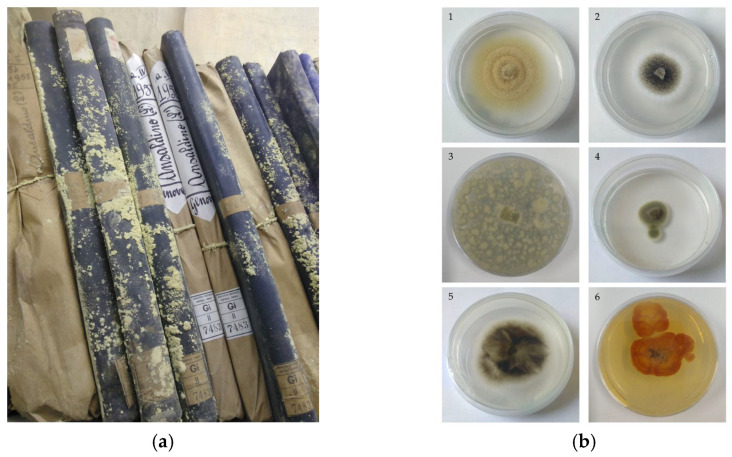
(**a**) Book collection affected by fungal proliferation. (**b**) Pure cultures of fungi isolated from paper documents: (1) *Aspergillus* sp. section Circumdati; (2) *Aspergillus* sp. section Nigri; (3) *Penicillium* sp.; (4) *Cladosporium* sp. (5) *Alternaria alternata*; (6) *Aspergillus* sp.

Biodeterioration is a process of degradation instigated by fungi, bacteria, insects, rodents and other biotic agents that feed on organic materials, known as “biodeteriogens” [19]. When dealing with biodeterioration of paper, if a legal process could be undertaken against all biodeteriogens inhabiting archives and libraries, fungi would undoubtedly be declared guilty [19,23,25]. They are the most significant cellulase-producing micro-organisms and, due to being provided with a rich set of other degradative enzymes, they are able to proliferate by decomposing a variety of organic materials typically constituting historical collections, such as paper, leather, parchment, etc., with serious consequences for the preservation of artworks [19,32] (Figure 1a). Not by chance, fungi were the main target of the antimicrobial tests reported in the publications reviewed here (Figure 1b). *Aspergillus*, *Chaetomium*, *Cladosporium* and *Penicillium* were among the most recurrent investigated genera [10,11,12,13,22,24,28,29], whereas bacteria and yeasts were considered to a lesser extent [11,22,24,26,27,28], and insects were investigated only by Menicucci et al. [28].

Lavin et al. [23] studied the degradative action carried out by *Scopulariopsis* sp. and *Fusarium* sp. on a variety of archival documents, including photographs, books and maps. They examined fungal bioadhesion and biofilm formation via scanning electron microscopy (SEM), revealing a severe fungal attack that caused a significant decrease in the substrate pH value. They also propose the use of EOs to reduce fungal proliferation, due to encouraging results obtained *in vitro*, but did not test them on paper supports.

For a first *in vitro* evaluation of EO antimicrobial efficacy, the most commonly used antimicrobial tests were disk diffusion [11,13,22,29] and the micro-atmosphere method [10,23,28,29]. This latter approach is probably the most recommended method, as it does not involve direct contact between the target micro-organism and the tested substance [30]. In this way, the growth inhibition exerted by an EO is only referable to its volatile phase, which is the fraction playing the major role in EO bioactivity [23]. Moreover, it is also more suited to mimicking an on-site scenario at laboratory scale, as direct application of these substances to contaminated materials should be avoided to ensure non-invasive treatment.

In regard to other types of tests, despite some case studies discussing books and historical manuscripts disinfected with EOs [10,11,24,27], as well as other publications showing experimental setups meant to closely reflect real on-site conditions [12,25], there is still a long way to go until a safe and effective EO application in this field is achieved.

The most frequently tested EOs were thyme [11,13,23,24,27] and oregano [13,22,23,27,29]. Considering the monoterpenes, a special mention is given to linalool, whose use is a matter of debate. It was tested by Rakotonirainy and Lavédrine [10] with successful results for paper, and later investigated again by the same research group, who reported significant damage to other archival materials, i.e., silver–gelatine photographs and leather bookbindings [33]. The authors concluded that a careful evaluation must be performed in the selection of one disinfection substance over another, as the response of a material to a treatment can significantly vary depending on the material used. As many collections do not only include paper-based objects, this aspect that should not be neglected.

## 4. Book Disinfection with EOs

In ancient times, leaves from medicinal plants were placed across book pages to preserve manuscripts from woodworms (“Del furore d’aver libri” by Gaetano Volpi, 1756). There is also evidence that during the XXth Century, plant essences were applied in the form of hydro-alcoholic solutions to books in historical collections in order to prevent mold proliferation. The first scientific publication attesting such a method of conservation involved the use of thymol [21]. The authors affirmed that thymol was commonly used to soak paper and picture frames, being a fungicide remedy. This type of direct-contact treatment was performed on a collection of maps and prints in a 3-year exhibition, which took place from 1980 to 1983, at the end of which the prints were found to have yellowed. Interestingly, it was also noticed that greater yellowing occurred in those areas which had been more light exposed. The authors set up an experimental framed print to test its response to thymol. They observed that in presence of light and oxygen, thymol was photo-oxydized, as its crystals discolored after few days. The same yellowing was observed on Whatman paper treated with thymol, as well as on the polymethyl methacrylate sheets used for glazing the prints. These sheets were also found to absorb the crystals of thymol. The authors concluded that thymol should not be used as fungicide agent on artworks undergoing exposition due to photo-oxidation-related damages, i.e., significant yellowing of different kinds of materials [21].

Although some recent papers still report direct-contact EO/VOC-based treatments [11,27], the indirect treatments seem to be more in agreement with a non-invasive approach. For instance, Rakotonirainy and Lavédrine proved the fungistatic action of linalool vapours on a book within a showcase. The book was previously inoculated with a mix fungal suspension and kept under controlled conditions for 21 days. No significant damages occurred to the material in terms of color alterations or pH acidification [10].

## 5. Structural Analysis of Paper

To detect any possible alteration of the material undergoing experimentation, mechanical, physical and optical properties should be monitored pre- and post-treatment. The most frequently investigated parameters include total color difference (ΔE), yellowness index and surface pH [10,11,12]. For example, Karbowska-Berent et al. [12] used tea tree oil to disinfect paper-based objects and examined the above-mentioned parameters, in addition to tear resistance, to evaluate its influence on paper. They recommend not using tea tree oil on paper-based documents because of the unacceptable alterations caused by this treatment in all samples.

In a recent publication of Benkovičová et al. [13], capsulated EOs were tested for their antifungal properties on a set of prototypes representing materials of significant interest for the cultural heritage sector. Whatman paper was chosen as standard reference for paper items, and color changes after treatment were detected by measuring the surface reflectivity. They found that the higher the EO concentration, the lower the registered reflectivity for all types of analyzed surface. Moreover, the color was not considerably altered by the lowest concentration used (10% of capsulated EOs).

When dealing with paper documents, another indicator of structural alteration to be considered is cellulose degree of polymerization (DP), which reflects the state of cellulose degradation [34]. This parameter is strictly related to the mechanical properties of a material. From a chemical point of view, cellulose depolymerization is mainly due to hydrolysis and oxidation of glycosidic bonds, resulting in loss of mechanical strength in paper [32,35,36]. Cellulose DP can be measured via viscometry or size-exclusion chromatography (SEC), as shown in Čabalová et al. [25]. However, both of these methods are destructive; therefore, their application in the field of cultural heritage is strongly limited for obvious reasons [37].

On the contrary, Fourier transform infrared spectroscopy (FTIR) can give structural information about paper-based items without damaging samples under investigation. The presence of components other than cellulose, which may be the case in EO treatment residues in the sample, creates different FTIR signals, which are clearly distinguishable from cellulose FTIR profile. This technique was used by Noshyutta et al. [11] to check the effect of different concentrations of tea tree, lavender and thyme EOs on paper samples.

## 6. EO-Based Technologies

Many investigations are currently focusing on the development and testing of new EO-based products, aiming to achieve improved over time efficacy and stability of substances that are inherently susceptible to quick degradation. Commercial products are usually aqueous solutions of active ingredients; thus, the weak hydrophilicity of EOs is an aspect of major concern for designing new systems [30]. In addition, those EO constituents showing major bioactivity are VOCs, i.e., molecules displaying short-term efficacy due to scarce stability. These are limiting factors hampering easy management and effectiveness, and in view of this, resorting to new technologies may represent a strategy to be explored.

Depending on the intended use, different systems are being experimented in multiple research areas, including nanoparticles [13], nanocapsules [8], liposomes [38], hydrogels [15], cyclodextrins [39] and others. With regard to the conservation of paper-based items, there are a few pioneering papers, which tested three different technologies/EO-based products with the aim of to achieving empowered efficacy.

Campanella et al. [26] combined cinnamon EO with psyllium and psyllium mixed with alginate, respectively, obtaining gel beads that showwed high rates of encapsulation and controlled EO release. By exploiting three naturally occurring substances, they obtained an innovative EO-based product that displayed extended shelf life and efficacy, which was meant to be tested in the protection of cellulosic cultural heritage objects. To evaluate the antimicrobial properties of these gel spheres, they performed a respirometric test on *Saccharomyces cerevisiae*, and results showed good levels of antimicrobial activity. Unfortunately, for now, there is no further investigation into such technology’s impact on cellulosic items that give full insights into its on-site applicability.

Another study by Benkovičová et al. [13] tested the use of capsulated EOs in nanoparticles. Specifically, they synthesized super-hydrophobic nanoparticles (SHNPs) loaded with three EOs (arborvitae, oregano and thyme EOs) to be tested on different material surfaces subjected to fungal attack. Experimental supports were paper (Whatman type), sandstone and whitewood; these supports were coated with SHNPs encapsulating EOs and inoculated with two particularly aggressive fungal strains (*Aspergillus fumigatus* and *Exophiala xenobiotica*) towards these substrates. They aimed to obtain a super-hydrophobic layer, protecting the materials’ surface from fungal proliferation and penetration. Very different responses to the treatment were observed depending on the material, as well as the type and concentration of EO, used. For example, increasing concentrations of thyme EO nanoparticles proportionally reduced fungal proliferation on paper, whereas for sandstone samples, the lowest concentration was the only effective concentration. They also underline the importance of defining safety thresholds of treatment to prevent undesired alterations in the original properties, as excessive concentrations may exert an antifungal effect and, at the same time, cause damage to the material. Once again, this study shows material-dependent results, highlighting the importance of a detailed investigation that takes into account multiple parameters to identify an effective and non-invasive treatment.

Finally, our previous study [28] reports the use of β-cyclodextrins and cocrystals as solids built with VOCs of thymol, carvacrol and eugenol, which were tested against some paper biodeteriogens. Two kinds of powdery products based on EO constituents were produced and showed different degrees of bioactivity towards a variety of biodeteriogens selected for the investigation, including fungi, a bacterium, a yeast species and a polyphagous insect that had recently spreading into the archives. Superior levels of efficacy were observed when using the carvacrol-based cocrystal, in terms of both antimicrobial and insect repellent activity. Although all tests were performed at the laboratory scale, a first pilot test was realized on Whatman paper deliberately inoculated with fungal suspensions of known concentration to assess the antifungal activity of the carvacrol-based cocrystal. Paper samples were exposed to the vapors released from the cocrystal, and a significant inhibition of the fungal growth was registered for those samples undergoing cocrystal treatment with respect to non-treated samples. These preliminary results show the efficacy of carvacrol release from a solid product, which was applied in a non-invasive way to a reference standard paper.

## 7. Conclusions

The use of aromatic plants and their extracts for the preservation of book heritage is historically documented. However, a research-driven approach to the testing of EOs and their main constituents on paper-based objects should be based on their chemical analysis via GC-MS techniques. As plant derived substances characterized by a high degree of complexity in their composition, EOs represent a unique source of versatile compounds, meeting the need for innovation in a sector where toxic chemicals are still largely used. Few recent publications report new EO-based products, ensuring prolonged efficacy over time and controlled release. This approach seems to be the most promising path to explore effective use of intrinsically unstable products. In addition, treatments ensuring indirect contact with the material should be preferred to direct treatments in order to avoid potential damages. The applicability of EO-based treatments to paper is far from being determined, as more investigation is required to define the safety threshold for an effective use of these substances based on the non-invasiveness and non-toxicity criteria. These two aspects should be the pivot around which upcoming research in this field rotates.

## Figures and Tables

**Table 1:** Key references dealing with essential oils (EOs)/volatile organic compounds (VOCs)/derived products for conservation of paper-based items.

EO/VOC/EO-Based Product	GC-MS Analysis	Target Species (Bacterium/Fungus/Yeast/Insect)	Test with EO/VOC/EO-Based Product	Analysis and Examined Paper Parameters	Reference
**Thymol**	-	*-*	Direct contact treatment: thymol alcoholic solution (7% *w/v*) used to impregnate a blotting paper to be placed behind a pencil drawing	Visual inspection	[21]
***Artemisia vulgaris* (armoise), *Peumus boldus* (boldo), *Eugenia caryophyllata* (clove), *Eucalyptus globulus* (eucalyptus), *Lavandula angustifola* (lavender), *Ravensara aromatica* (ravensare), *Malaleuca alternifola* (tea tree), *Thuja occidentalis* (thuya), or *Chenopodium ambrosioides* (wormseed)**	-	*Aspergillus niger, A. fumigatus, A. repens, Cladosporium herbarum, Penicillium frequentans, Trichoderma viride, Chaetomium globosum, Paecilomyces variotii, Stachybotrys atra*	Microatmosphere method (5, 35, 50, 60 µL in 90 mm Petri)	pH of the cold extract; diffuse reflectance factor (brightness); viscometric average degree of polymerisation (DPv) of cellulose	[10]
**1,8-cineole, eugenol, linalool, linalyl acetate,** **⍺** **+β** **thujone**			Inoculation of a mix fungal suspension on paper supports inserted into books and exposed to vapors of linalool (295 and 415 ppm), at 25 °C for 21 days, in sealed chambers		
***Pimpinella anisum* L. (anice), *Syzygium aromaticum* L. (clove), *Cuminum cyminum* L. (cumin), *Allium sativum* L. (garlic), *Laurus nobilis* L. (laurel), *Citrus sinensis* (L.) Osbeck (orange sweet) or *Origanum vulgare* L. (oregano)**	Yes	*Bacillus* sp., *B. polymyxa, B. cereus, B. thuringiensis, Enterobacter agglomerans, Streptomyces* sp.	Agar diffusion method	-	[22]
		*Aspergillus niger, A. clavatus*, *Penicillium* sp., *Fusarium* sp.			
***Origanum vulgare* L. (oregano), *Thymus vulgaris* L. (thyme)**	-	*Fusarium* sp., *Scopulariopsis* sp.	Microatmosphere method-10 µL of pure EOs	-	[23]
Lavender, tea tree, thyme	-	*Bacillus subtilis*	Disk-diffusion 0.031–0.063–0.125–0.25–0.5–1.0–2.5% (*v/v*)	Surface morphology (SEM) of colonized and non-colonized paper items	[11]
		*Aspergillus flavus, Eurotium chevalieri, Penicillium roqueforti, Trichoderma viride*	Fumigation of EOs on mimic paper samples—concentrations used: 0.125–0.25–0.5%	Total color difference (ΔE), whiteness index (W), yellowness index (Y)	
			Application of tea tree (0.25% *v/v*) in the leaf casting stage of a manuscript	tensile strength, elongation at break	
				FTIR-ATR	
***Thymus vulgaris*** (thyme)	-	*Bacillus cereus, B. licheniformis, Microbacterium aerolatum, Psychrobacillus psychrodurans, Staphylococcus epidermis, S. pasteuri, S. saprophyticus, S. succinus*	Tests on paper-Thyme essential oil microatmosphere (conc. 10% in DMSO) to treat two books	Dimensional and structural parameters (weight; thickness; bulk; air resistance; ash; pH; Kappa number; intrinsic viscosity)	[24]
		*Aspergillus niger, Chaetomium elatum, C. globosum, C. murorum, Myxotrichum deflexum, Penicillium spinulosum, Rhodotorula mucilaginosa*		Mechanical parameters (Stretch; tensile index; TEA; burst factor; tear factor; folding endurance; ZSFS)	
				Optical parameters (R457; yellowness; L *; a *; b *; ΔE *)	
Tea tree	-	Cladosporium cladosporioides, Penicillium spinulosum *, Trichoderma pseudokoningii	paper exposure to tea tree vapors (1–3 mL/l) after infection with *P. spinulosum*	Surface pH	[12]
				Total color difference (ΔE), difference in yellowing ΔRz	
				Tear resistance	
super-hydrophobic nanoparticles loaded with ***Thuja plicata*** (arborvitae), ***Origanum vulgare* L.** (oregano) or ***Thymus vulgaris*** (thyme) EO	-	Aspergillus fumigatus *, Exophiala xenobiotica	Disk-diffusion (EO mix with SHNPs at conc. 10–30–50% in EtOH)	Rr (ratio of reflectivity) spectral reflectivity measurements	[13]
			Test on Whatman paper (100 µL of EO/EO mix with SHNPs)		
Mix of linalyl acetate and citral	-	-	Aged and unaged paper samples exposed to vapors of linalyl acetate mixed with citral (1:1), RH = 75%, in a desiccator	Chemical parameters (Cellulose degree of depolymerization; content of saccharides and lignin)	[25]
				Mechanical parameters (tensile index)	
				Physical parameters (fiber length determination)	
Cinnamon EO-gel spheres	yes	*Saccharomyces cerevisiae*	Respirometric test	-	[26]
***Origanum vulgare*** (oregano) or ***Thymus vulgaris*** (thyme)	-	*Staphylococcus epidermidis*	Thyme EO (0.75% *v/v*) nebulized immediately after the inoculation on agar plates or paper sheets	-	[27]
		*Alternaria alternata*	Application of thyme EO (0.75% *v/v*) on a contaminated book cover by means of EO impregnated contact sheets		
		*Rhodotorula mucilaginosa*			
β-cyclodextrins and cocrystals entrapping carvacrol, thymol or eugenol	Yes	*Bacillus* sp.	Micro-atmosphere method	-	[28]
		*Alternaria alternata, Aspergillus* sp. *(section Nigri), Cladosporium* sp., *Trichoderma orientale*	Whatman paper exposed to vapors of carvacrol-based cocrystal (30 mg) after fungal infection		
		*Metschnikowia* sp.	Olfactometer bioassay		
		*Lasioderma serricorne*			
***Eucalyptus globulus*** (eucalyptus), ***Cymbopogon citratus*** (lemongrass), ***Origanum vulgare*** (oregano), ***Mentha piperita*** (peppermint), or ***Rosmarinus officinalis*** (rosemary)	Yes	*Aspergillus fumigatus, Cladosporium cladosporoides, Penicillium chrysogenum*	Disk diffusion (15 µL of pure EOs)	-	[29]
			Vapor phase (15 µL of pure EOs)		
			Mixture of oregano, lemongrass and pepper mint EOs (1:1:1) in vapor phase (15 µL) in Petri dishes used to treat historical paper samples (1 cm^2^) inoculated with fungal suspension		

* Target of treatment with EO/VOC/EO-based product for those studies not testing all micro-organisms listed.
